# The impact of type 2 diabetes on bone metabolism

**DOI:** 10.1186/s13098-017-0278-1

**Published:** 2017-10-19

**Authors:** Claudia Pinheiro Sanches, Andre Gustavo Daher Vianna, Fellype de Carvalho Barreto

**Affiliations:** 1 Curitiba Diabetes Center, Division of Endocrinology, Hospital Nossa Senhora das Graças, Rua Alcides Munhoz, 433, 4° andar, Mercês, Curitiba, Paraná ZIP Code: 80810-040 Brazil; 2 Pontifical Catholic University of Parana, Rua Imaculada Conceição, 1155 , Bloco Medicina, Prado Velho, Curitiba, Paraná ZIP Code: 80215-901 Brazil; 3 Division of Nephrology, Department of Internal Medicine, Federal University of Paraná, Rua General Carneiro 181, Alto da Gloria, Curitiba, Paraná ZIP Code: 80060-900 Brazil

**Keywords:** Type 2 diabetes, Bone metabolism, Bone mineral density, Fracture

## Abstract

Diabetes complications and osteoporotic fractures are two of the most important causes of morbidity and mortality in older patients and share many features including genetic susceptibility, molecular mechanisms, and environmental factors. Type 2 diabetes mellitus (T2DM) compromises bone microarchitecture by inducing abnormal bone cell function and matrix structure, with increased osteoblast apoptosis, diminished osteoblast differentiation, and enhanced osteoclast-mediated bone resorption. The linkage between these two chronic diseases creates a possibility that certain antidiabetic therapies may affect bone quality. Both glycemic and bone homeostasis are under control of common regulatory factors. These factors include insulin, accumulation of advanced glycation end products, peroxisome proliferator-activated receptor gamma, gastrointestinal hormones (such as the glucose-dependent insulinotropic peptide and the glucagon-like peptides 1 and 2), and bone-derived hormone osteocalcin. This background allows individual pharmacological targets for antidiabetic therapies to affect the bone quality due to their indirect effects on bone cell differentiation and bone remodeling process. Moreover, it’s important to consider the fragility fractures as another diabetes complication and discuss more deeply about the requirement for adequate screening and preventive measures. This review aims to briefly explore the impact of T2DM on bone metabolic and mechanical proprieties and fracture risk.

## Background

Type 2 diabetes mellitus (T2DM) is associated with an increased risk of fracture, although bone mineral density (BMD) is unaffected or even higher in diabetic patients [1]. The reasons involve likely a combination of features, including the duration of disease, inadequate glycemic control, greater risk of falling as a consequence of hypoglycemia, osteopenia, impairment of bone quality, and side effects of medication, which could lead to a higher risk of bone fragility and fractures [1].

Unfortunately, there is little scientific knowledge approaching the impact of diabetes and of most anti-diabetic treatments on bone quality and fracture risk. Thus, this review aims to briefly explore the impact of T2DM on bone metabolic and mechanical proprieties and fracture risk. Moreover, an accompanying review about the pros and cons of the available pharmacologic treatments for T2DM on bone mineral density and risk of fractures in humans is provided in this issue of *Diabetology & Metabolic Syndrome* by Vianna et al. (doi:10.1186/s13098-017-0274-5).

## T2DM and higher risk of bone fracture

The prevalence of T2DM has augmented with the growth in obesity epidemics, mainly because of the lifestyle changes imposed by the modern life. Patients with poorly controlled T2DM are at increased risk for diabetic complications, including macrovascular disease, retinopathy, nephropathy, and neuropathy. Recently, an increased risk of fragility fractures has been recognized as another significant diabetes complication [2]. According to Rotterdam study, individuals with T2DM have a 69% increased risk of having fractures when compared with healthy controls. Paradoxically, T2DM subjects had greater BMD of the femoral neck and lumbar vertebrae [3]. The discrepancy between BMD and fracture incidence observed in T2DM patients could be attributed to a frailer bone material causing failure at lower stress or to the impaired biomechanical skeletal properties [4]. Osteoporosis is one of the most important causes of reduced bone mineral density, and it is estimated to affect 200 million women worldwide. It accounts for more than 8.9 million fractures annually in women over age 50 [5]. T2DM and osteoporosis are both chronic diseases that may coexist and progressively increase in prevalence and are boosted by aging [6, 7].

It has been observed that T2DM negatively affect bone strength regardless of BMD [1, 8]. The greater risk of fracture is demonstrated by the health, aging and body composition study, where the relative risk (RR) of fracture was 1.64 (95% CI 1.07–2.51) in those with diabetes compared to those without, even after adjustments for hip BMD and additional risk factors for fracture [9]. Typically, T2DM patients have a normal BMD, so this increased risk is probably due to abnormalities in bone material strength and bone biomechanical quality [10]. Some cross-sectional studies in T2DM patients using high-resolution peripheral quantitative computed tomography (HR-pQCT) and magnetic resonance imaging (MRI) revealed quality defects in both cortical and trabecular bone [10]. Farr et al. [10] by assessing bone quality with HR-pQCT in 30 postmenopausal T2DM patients at the distal radius and distal tibia, found that the cortical thickness in T2DM subjects was lower than in controls. Moreover, bone microindentation testing displayed lower bone material strength (BMS) in post menopausal women with T2DM compared to those without diabetes [11]. Patsch et al. [12], investigated bone microarchitecture changes in postmenopausal T2DM patients with or without fractures at radius and tibia by using dual-energy X-ray absorptiometry (DXA) and HR-pQCT. They concluded that T2DM patients with fractures had higher pore-related deficits and a greater cortical pore volume than diabetic patients without fractures. Cortical defects often accompanied the impaired mechanical properties, such as increased failure load and low bone bending strength, that led to a reduction in overall bone strength and increase in fracture risk [13]. It seems like that bone trabecular and cortical microarchitecture are both deranged in T2DM and may contribute to bone fragility [11, 14]. Bone remodeling decreases, as demonstrated by histomorphometric analysis of bone, which is an additional contributor to the increased the risk of fragility fractures in T2DM patients [15, 16].

Patients with T2DM have an elevated risk of all clinical fractures, particularly African-American and Latino populations [16]. Ageing, prior fracture, corticosteroid use, longer duration of diabetes and poor glycemic control are all contributory factors. Complications comorbidities and diabetic complications such as sensory neuropathy and visual impairment imply in greater risk of falling [4]. Moreover, falling risk may also be associated, at least partially, to increased rates of hypoglycemia, postural hypotension, and vascular disease, contributing to increased risk of fragility fracture [17–19].

## Cross-talk between glucose homeostasis and bone metabolism

Recent evidence of common regulatory control of both glycemic and bone homeostasis enables to recognize the intimate relationship between these two entities and similarly the likelihood of antidiabetic agents to impact the bone quality. The shared regulatory control includes accumulation of advanced glycation end products (AGEs), insulin, insulin-like growth factor-1 (IGF-1), peroxisome proliferator-activated receptor gamma (PPARγ), the incretin hormones like glucose-dependent insulinotropic peptide (GIP), glucagon-like peptide 1 and 2 (GLP-1 and GLP-2), the bone-derived hormone osteocalcin and sclerostin.

The impact of vitamin D levels on glycemic control and bone mineral density in postmenopausal women with T2DM have also been studied [20]. Vitamin D [25 (OH) D3] plays a fundamental role in bone metabolism and might impact the development and control of diabetes [21, 22]. Some studies have reported an inverse relationship between HbA1c levels and serum levels of 25 (OH) D3 [22], while others have found that 25 (OH) D3 supplements improve glucose control in T2DM [22, 23]. Physiologically, vitamin D seems to stimulate the expression of the insulin receptor. Therefore vitamin D deficiency might be associated with insulin resistance [24]. Recently, Perez-Diaz et al. [20] have attempted to evaluate the impact of vitamin D levels on glycemic control and bone metabolism. They failed to demonstrate a clear relationship between 25 (OH) D3 levels and glucose control or osteoporotic fractures, even though reported that patients with poor glycemic control had lower 25 (OH) D3 levels than controls.

### Advanced glycation end products (AGEs)

The hyperglycemia affects both cellular and extracellular bone matrix. The presence of glucose induces the formation of intermediate products containing highly reactive dicarbonyls, which ultimately leads to the production of irreversible accumulation of advanced glycation end products compounds [25], from a non-enzymatic glycation process [26]. The congeries of AGEs determines the formation of defective collagens and reactive oxygen species (ROS), inducing structural changes in bone through posttranslational modifications [27]. At the organic bone matrix, these reactions may lead to impaired bone strength [28, 29]. Higher levels of circulating AGEs are reported to increase fracture risk [30].

AGEs bind to the receptor for AGE (RAGE), which is a member of the immunoglobulin superfamily, and it is the AGE-RAGE interaction that mediates generation of ROS, vascular inflammation, macrophage and platelet activation, and stimulates the migration of inflammatory cells [31]. All these reactions contribute to the development and progression of diabetic macro- and microangiopathy and result in a more brittle bone with reduced strength and less ability to deform before fracturing [32].

**Fig. 1:**
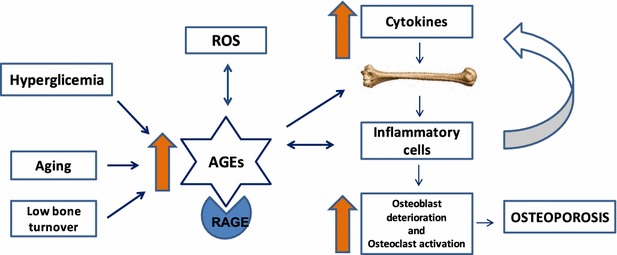
The relationship between the accumulation of AGEs within the bone. Increased oxidative stress, high glycemic levels, ageing and reduced bone turnover are the main contributors to increased formation and accumulation of AGEs in bone. They induce an inflammatory process that results in activation of osteoclastogenesis, osteoblast dysfunction and accelerated development of the osteoporosis process (Adapted from Sanguineti et al. [33])

Immune cells also express RAGE and incite activation of nuclear factor kappa-light-chain-enhancer of activated B cells (NF-κB), a central transcription factor of the immune and inflammatory response [31]. The AGE-RAGE linkage in immune cells results in upregulation of inflammatory cell adhesion molecules and chemokines, releasing, even more, RAGE ligands, and sustaining the inflammatory tissue response, modulating the response of activated macrophages to increase the damaging signals in the tissues and suppressing the repair and remodeling reactions [31]. In a microenvironment with incremental inflammatory cytokines, AGEs may induce osteoclastogenesis and osteoblast dysfunction, which may ultimately result in the development of osteoporosis (Fig. 1) [33]. Pentosidine, the most studied AGE in T2DM patients, accumulates in the cortical and trabecular bone and negatively impact the bone strength and probably leads to functional changes in osteoblasts and the bone mineralization process [34, 35].

The outcome of these reactions is reduced strength and impaired biomechanical properties of both trabecular and cortical bone, including disturbance in osteoblast function and attachment to collagen matrix, damaging the healthy development [30, 35–37].

### Insulin and IGF-1

Insulin is an anabolic hormone which acts on bone tissue through its receptors (IRS-1 and IRS-2) expressed by osteoblasts, stimulating bone formation. Insulin increases osteoblast proliferation and promotes collagen synthesis. In the same way, insulin growth factor-1 (IGF-1) acts increasing osteoblast recruitment and bone matrix deposition and diminishing collagen degradation. Studies have exhibited a positive correlation between IGF-1 and BMD, and a negative correlation with hip and vertebral fracture [38, 39].

### The peroxisome proliferator-activated receptor gamma (PPARγ)

**Fig. 2:**
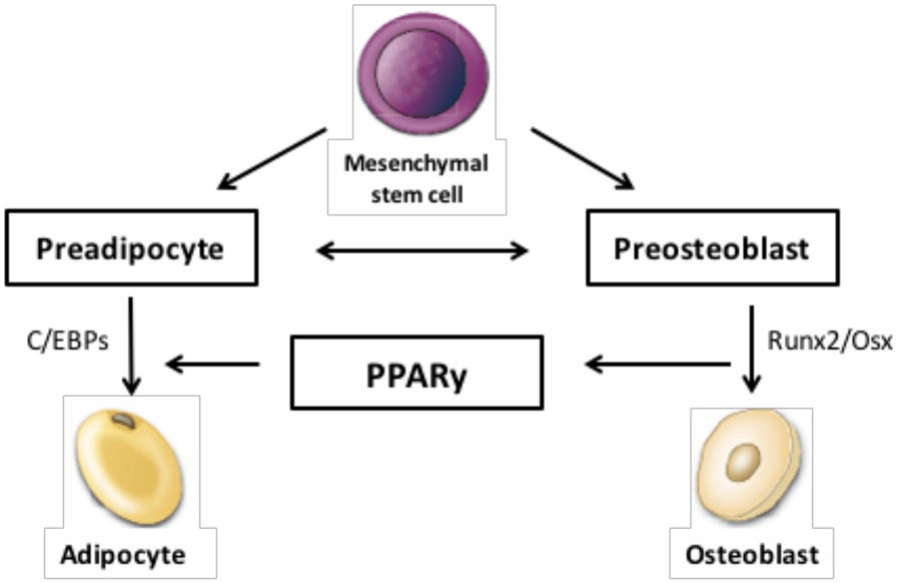
PPAR regulates mesenchymal cell differentiation. PPAR induces adipogenesis and suppresses osteoblastogenesis, by inhibiting Runx2 function, resulting in a reduction of osteoblasts in the bone marrow. *C/EBPs* CCAAT/enhancer binding protein*s*, *Osx* osterix, *Runx2* runt-related transcription factor 2 (Adapted from Kawai et al. [43])

The PPARγ protein is an essential regulator of lipid, glucose, and insulin metabolism. There are two isoforms in humans, PPARγ1 and PPARγ2. PPARγ1 is expressed in a variety of cell types, including osteoclasts, promoting their differentiation and bone resorption [40]. The PPARγ2 expression restricts to cells of adipocytic lineage [41]. In bone, PPARγ2 plays a significant role in the regulation of mesenchymal cell (MSC) differentiation toward osteoblasts and adipocytes. When this isoform is activated, cells of osteoblast lineage are converted to terminally differentiated adipocytes, disturbing the delicate balance between bone marrow adipocytes and osteoblasts (Fig. 2) [42].

### The role of enteric hormones

The glucose-dependent insulinotropic peptide (GIP) and the glucagon-like peptides 1 and 2 (GLP-1 and GLP-2) are hormones released by gut enteroendocrine K-cells in the duodenum and proximal jejunum and from L-cells located in the distal ileum and colon, respectively [44]. GIP and GLP-1 are secreted just after nutrient ingestion. They are already released into circulation in their active hormonal form and bind to a specific G protein-coupled receptors present in several cells and target tissues. Both hormones have their bioactivity limited by rapid degradation and inactivation by the enzyme dipeptidyl peptidase-4 (DPP-4), which is present in plasma and expressed in most tissues [45]. The incretin hormones (GIP and GLP-1) stimulate insulin release from β-cells to inhibit glucagon production by the α-cells [46]. Incretin receptors are also expressed in both osteoblasts and osteoclasts. These nutritional hormones are recognized to be significant in bone turnover since as soon as a meal is ingested, bone resorption is suppressed [47, 48]. In times of energy and nutrient excess, the balance is tipped for bone formation, whereas while energy and nutrient are lacking, bone resorption increases [47]. GIP and possibly GLP-1 and GLP-2 may link nutrient ingestion to suppression of bone resorption and stimulation of bone formation [49]. Studies indicate that GLP-2 may affect bone remodeling by disassociating bone resorption and bone formation [50], acting mainly as an antiresorptive hormone [50], while GIP can act both as an antiresorptive and anabolic hormone [49, 51].

### Bone turnover markers: focus on osteocalcin

An additional approach to evaluate the impact of diabetes on bone metabolism is to assess the serum markers of bone turnover (BTM), particularly the formation markers osteocalcin (OC) and amino-terminal propeptide of procollagen type 1 (PINP), which are decreased in these patients [52, 53]. Shu et al. [54] investigated structural and biochemical skeletal parameters in T2DM patients and shown that postmenopausal women with T2DM had lower levels of bone formation markers when compared to controls, while their bone structure was not modified. They found lower OC and PINP levels in diabetic subjects, and these levels correlated inversely with glucose levels and fat mass. This concept supports the idea that biochemical indices of bone formation are lower in T2DM patients than in controls. Moreover, the resorption marker CTX (serum C-terminal telopeptide from type 1 collagen) is shown by some authors to be reduced in T2DM individuals [52, 55], while other revealed no difference [56].

Interestingly, OC seems also to have a role in energy metabolism. In its undercarboxylated form, OC stimulates insulin secretion and enhances insulin sensitivity in both adipose and muscle tissue. An inverse association between OC and metabolic syndrome has been demonstrated, suggesting that reduced levels of osteocalcin may impact in the pathophysiology of T2DM [57, 58]. Consequently, the skeleton has been considered a new endocrine organ that participates and influences glucose homeostasis.

### The Wnt/ß-catenin pathway

**Fig. 3:**
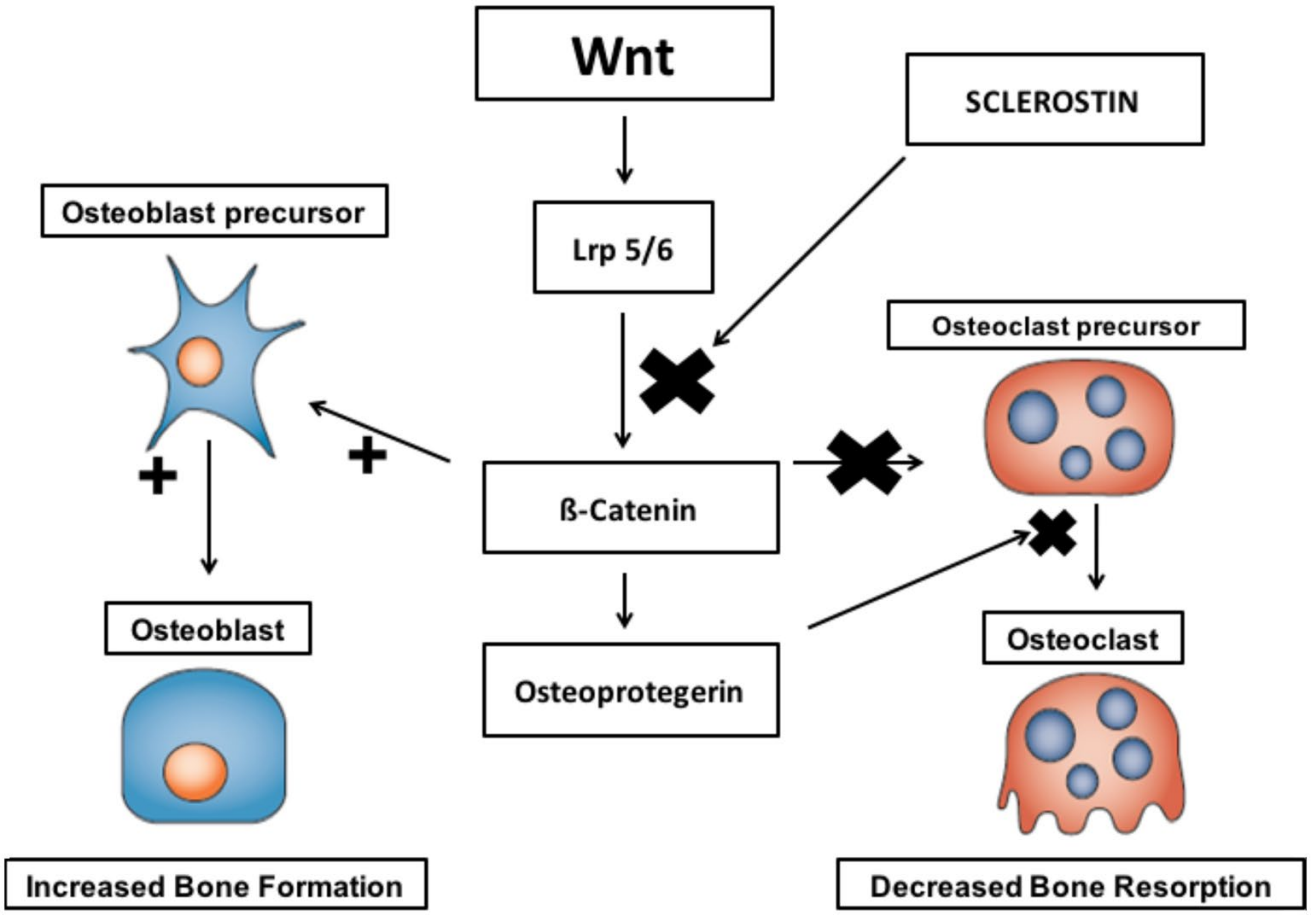
Canonical Wnt signaling and bone remodeling. T2DM patients present a greater amount of sclerostin, which blocks the Wnt pathway and inhibits osteoblastogenesis. *Lrp* lipoprotein receptor-related protein (Adapted from Canalis et al. [59])

Sclerostin is another regulator of bone metabolism and is expressed by osteocytes. It inhibits the Wnt/ß-catenin pathway by binding to low-density lipoprotein receptor-related protein (LPR) 5 or 6 and negatively regulates bone formation [59]. The Wnt/ß-catenin pathway induces osteoblastogenesis and thereby enhances bone formation. Canonical Wnt signaling suppresses osteoclastogenesis by inducing osteoprotegerin, and, also, suppresses bone resorption by an osteoprotegerin-independent mechanism acting directly on osteoclast precursors. The dual effect of Wnt on cells of the osteoblast and osteoclast lineage results in an increase in bone mass. So, when sclerostin bind to Wnt co-receptors, inhibition of osteoblastogenesis and bone formation occurs (Fig. 3) [59]. Patients with T2DM have higher serum levels of sclerostin, which are associated with increased risk of vertebral fractures. Studies also show that sclerostin levels is directly related to both duration of T2DM and glycated hemoglobin, and inversely related to levels of bone turnover markers [52, 60].

### The impact of vitamin D

The hyperglycemia seems to play a major role on the vitamin D-calcium axis through impairment renal calcium absorption [61]. High glycemic levels contribute to the reduced number of 1,25(OH)_2_D3 (1,25-dihydroxy vitamin D) receptors on osteoblasts and limit the ability of the osteoblast to synthesize osteocalcin in response to 1,25(OH)_2_D3 [61]. However, the vitamin D performance in affecting T2DM and fracture risk is currently uncertain [31].

### Summary

As reviewed in the topics above, several direct and indirect mechanisms in T2DM may affect the bone metabolism and quality, as well the risk of fractures. Table 1 review and summarize the effects of type 2 diabetes on bone.

**Table 1:** Summary of the mechanisms by which T2DM negatively affects the bone

	Mechanisms	Effects on bone
AGEs	Osteoclastogenesis and osteoblast dysfunction [28]	Low bone quality [29, 30] Increased risk of fragility fracture [28]
Insulin and IGF-1	Increases osteoblast proliferation and promotes collagen synthesis [38]	Negative correlation with hip and vertebral fracture [39]
PPARγ	Differentiate MSC into adipocytes [42]	Suppression of osteoblastogenesis [43]
Enteric hormones (incretins)	Energy intake releases GIP and GLP-2 [47–50]	Low incretin levels decrease bone formation and augment resorption [47–50]
Osteocalcin	Low levels in T2DM [54]	Low levels decrease bone formation [57, 58]
Wnt/B-catenin pathway: sclerostin	High levels in T2DM [59]	High sclerostin levels increase bone resorption [59]
Vitamin D3	Low levels in T2DM [31] Reduction of 1,25(OH)_2_D3 receptors [61]	Reduction of osteocalcin synthesis [61]

The indirect and direct effects of compromised glucose/insulin metabolism on bone induces a decreased bone turnover, a reduced bone quality and an augmented risk of fractures

*AGEs* advanced glycation end-products, *IGF*-*1* insulin-like growth factor-1, *PPARγ* peroxisome proliferator-activated receptor gamma, *MSC* mesenchymal stem-cells, *GIP* glucose-dependent insulinotropic polypeptide, *GLP*-*2* glucagon-like peptide-2, *T2DM* type 2 diabetes mellitus, *1,25(OH)*_*2*_ *D3* 1,25 dihydroxy vitamin D

## Conclusion

Patients with T2DM have an augmented risk for fragility fractures, not predictable by BMD measurements. This higher risk is probably multifactorial. Despite these features, there are no current recommendations regarding routine screening or initiation of preventative medications for osteoporosis in patients with diabetes. Adequate glycemic control prevents this risk and reduces the micro-and macrovascular complications, which consequently, can contribute to diminish the production of AGE’s, reduce the vascular damage in the bone tissue and lessen the risk of falls. As reported, bone and energy metabolism are closely related, and this connection occurs since the differentiation of adipocytes and osteoblasts from the same mesenchymal stem cells. In hyperglycemic patients, bone formation decreases and all mechanisms described so far contribute to the poorer bone formation and quality, increasing fracture risk. Currently, it is essential to consider the fragility fractures as an additional diabetes complication, recognize the diabetes bone disease as a specific pathology, and discuss more deeply about the requirement for adequate screening and preventive measures.

## Abbreviations

AGEs: advanced glycation end products
BMD: bone mineral density
BMS: bone material strength
BTM: bone turnover markers
CTX: C-terminal telopeptide of type 1 collagen
DPP-4: dipeptidyl peptidase-4
DXA: dual-energy X-ray absorptiometry
GIP: glucose-dependent insulinotropic peptide
GLP-1: glucagon-like peptide 1
GLP-2: glucagon-like peptide 2
HR- pQCT: high-resolution peripheral quantitative computed tomography
IGF-1: insulin-like growth factor-1
IRS-1: insulin receptor substrate-1
IRS-2: insulin receptor substrate-2
LPR: low-density lipoprotein receptor-related protein
MRI: magnetic resonance imaging
MSC: mesenchymal cell
NF-κB: nuclear factor kappa-light-chain-enhancer of activated B cells (NF-κB)
OC: osteocalcin
P1NP: amino-terminal propeptide of procollagen type 1
PPARγ: peroxisome proliferator-activated receptor gamma
RAGE: AGEs’ receptors
ROS: reactive oxygen species
RR: relative risk
T2DM: type 2 diabetes mellitus
1,25(OH)_2_D3: 1,25-dihydroxy vitamin D
25(OH)D3: vitamin D
